# The evolvement of sexual and reproductive health policies in Korea

**DOI:** 10.4069/kjwhn.2021.11.30.1

**Published:** 2021-12-13

**Authors:** Ju-Hee Nho

**Affiliations:** College of Nursing, Jeonbuk Research Institute of Nursing Science, Jeonbuk National University, Jeonju, Korea

## Introduction

Sexual health means being physically free from sexual problems, feeling proud, and psychologically comfortable as a sexual being. It refers to a state of physical, mental, and social well-being related to sex. According to the World Health Organization (WHO), sexual health is fundamental to the overall health and well-being of individuals, partners, and families, and it contributes to the social and economic development of communities and countries. Not only does sexual health encompass a positive and respectful approach to sex and sexual intercourse, but it also requires the possibility of a pleasurable and safe sexual experience free from coercion, discrimination, and violence [1].

Reproductive health is related to the well-being of the reproductive system and processes related to reproduction [2]. Reproductive health was embodied in the International Plan of Action adopted at the International Conference on Population and Development in Cairo in 1994. Recently, the concept of sexual and reproductive health (SRH) was summarized based on the WHO’s Constitution, which defined SRH as “a state of physical, emotional, and social well-being in all aspects of SRH, not merely the absence of disease, disability, or infirmity” [3].

## Changing perspectives on sexual and reproductive health

Recently, reproductive health has gained increasing importance as low birth rate continues to increase. SRH is not only directly linked to gender equality and women’s well-being; but it is also intrinsically important when considering the role of economic development and sustainability in the future society, further affecting the health of mothers, infants, children, and adolescents [3]. In this respect, reproductive health is recognized as a right, and its management is important from the point of view of the life cycle.

In South Korea (hereafter Korea), SRH was historically limited to maternal and child health within the population and family planning policies framework, focusing on pregnancy, childbirth, and childrearing. In the 1970s and 1980s, strong international population policies were implemented, and discussions about population control took place. In Korea, although the goal of population suppression was achieved early, the birth control policy was pursued for a considerable period of time and finally discontinued in 1996, with the aim of improving the population quality. As the fertility rate fell sharply in the 2000s, the government implemented policies encouraging childbirth and childcare support [4]. However, as long-standing policies regarding population continued to view the female body as a tool for childbirth [5], policies promoting fertility were limited in their impact.

At the Cairo Conference in 1994, the population policy emphasized the concrete life of human beings instead of quantitative control of the population and confirmed that reproductive rights are human rights. To improve women’s status and empowerment, the following goals, to be pursued by 2015, were resolved: reduction of infant/child/maternal mortality rate, universal education, and universal access to reproductive health services, including family planning. The 1995 Beijing Congress recognized the key role of women in socioeconomic development and recommended ensuring reproductive health and rights without unwanted pregnancies/births [4]. That is, it recommended that the concept of right to SRH should be guaranteed as an individual’s basic right by the state in consideration of the suitability of SRH, based on the international human rights norms, since it is important for both men and women. Additionally, the global respectful maternity care movement [6] should be expanded and interpreted from the perspective of ensuring the right to fertility that supports the recovery of de-medicalized childbirth and the strengthening of the family’s health capacity, allowing people to enjoy human rights of childbirth.

## Policy direction for sexual and reproductive health in Korea

In 2005, South Korea enacted the Low Fertility and Aging Society Basic Act and began policy intervention, and the fourth low fertility/aging society policy roadmap [7] has been announced for 5 years from 2021. Compared to the first, second, and third master plans in the past, the population policy paradigm has changed. The fourth master plan aims for “a sustainable society where all generations are happy together” as the vision, by recognizing that the fertility rate or population phenomenon is not a result of state-led regulation and control, but that of individual’s social adaptation and choice. Furthermore, it clarified the direction to focus on structural change at the individual, family, community, and social levels [7]. In the fourth basic plan, “a society where we work together and care for each other” is presented as a strategic policy focus to counteract low fertility. It is paying attention to the “guarantee of sexual and reproductive rights throughout life” and “institutional acceptance of diverse families” were raised as policy tasks [7]. The specific future directions for achieving this are as follows:

1) Shifting the policy paradigm from pregnancy and childbirth-centered health to universal health for both men and women to increase the fertility rate.

2) Promote healthy gender awareness and create a culture that is safe from gender violence by guaranteeing the right to self-determination in an equal relationship where men and women respect each other.

3) Strengthening social responsibility for health management and disease prevention, safe pregnancy, and childbirth according to individual life cycle.

4) Reorganizing the legislation so that the health of women, infants, and children can be guaranteed throughout their lives, and preparing a comprehensive plan for sexual reproductive rights focusing on gender and human rights.

The government should implement policies not only supporting health services for pregnant women but also supporting universal health coverage from adolescence to old age, such as sexual health, contraception, cancer prevention with the expansion of the current human papillomavirus vaccination program to males, decreasing sexually transmitted infections, counter-acting gender-based violence, as well as supporting menstrual health. Moreover, policies should be designed to adopt the normative and pragmatic perspectives of sexual health and reproductive rights.

## Nurses’ role within sexual and reproductive health evolvement in Korea

Nurses in the field of maternal/women’s health can play an important role in SRH management. International models that outline a nursing education curriculum for nurturing SRH health experts as well as job training and maintenance education to improve communication skills in sexual counseling situations with participants [8,9] should be implemented. Additionally, as women’s health is currently not included as a specialty field in advanced practice nursing in Korea [10], increasing the number of midwives and expanding their roles may be a realistic and effective mode in promoting SRH. Additionally, interdisciplinary cooperation for a person-centered response to SRH should be strengthened, and policy knowledge based on women’s experiences should be created. As well-informed advocates for SRH and social responsibility, Korean nurses and midwives in maternal/women’s health can be a bridge and sounding board for SRH to become a platform toward a sustainable society where all generations are happy.
